# Bilateral adhesive capsulitis following influenza vaccination: A case report

**DOI:** 10.1002/ccr3.3072

**Published:** 2020-07-05

**Authors:** Austin R. Thompson, Erik R. Ensrud

**Affiliations:** ^1^ Department of Orthopaedics and Rehabilitation Oregon Health & Science University Portland Oregon USA

**Keywords:** adhesive capsulitis, influenza vaccination, shoulder, shoulder injury, vaccine

## Abstract

Bilateral shoulder adhesive capsulitis may develop in association with the administration of an influenza vaccine. Vaccine administration should utilize proper technique to avoid injection into the shoulder capsule.

## Graphical abstract

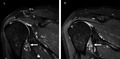

## 1 BACKGROUND

We describe a report of bilateral shoulder adhesive capsulitis developing in association with the administration of an influenza vaccine. This presentation of bilateral adhesive capsulitis of the shoulders following influenza vaccine is suggestive of an immune‐mediated response. Careful administration of vaccines to avoid injections into the shoulder capsule is suggested.

Influenza (flu) vaccinations are common, with 45% of all adults and 68% of adults older than 65 years receiving a vaccine in the 2018‐2019 flu season.^1^ Local adverse reactions to vaccinations most commonly include redness, swelling, and pain at the injection site. There are reported cases of shoulder injury related to vaccine administration (SIRVA).^2^, ^3^, ^4^, ^5^, ^6^, ^7^ Poor injection technique may result in certain shoulder‐related adverse events, such as bursitis or tendonitis. In addition, the vaccine composition may induce an immune response in tissues surrounding the shoulder.^4^ Current literature suggests that the immune response remains localized to the limb in which the injection was administered. However, we present a patient that developed bilateral adhesive capsulitis (frozen shoulder) after receiving an influenza vaccine, consistent with an immune response targeting the shoulder capsule synovium.

## 2 CASE PRESENTATION

A 64‐year‐old male patient was referred to clinic for a 6‐month history of bilateral shoulder pain. The patient denied any particular exertion or exercise in the days prior to the onset of pain. However, he reported receiving an influenza vaccine high up on the shoulder of the left arm a few days prior to waking up one morning with mild left shoulder soreness. He developed a similar soreness in the right shoulder a couple days later. The patient reported that he experienced pain in both shoulders with active range of motion. Specifically, he experienced pain in the right lateral posterior deltoid with reaching behind his back and shoulder abduction with his right arm. He also reported pain in the left anterior deltoid while reaching behind his back and across his body with his left arm. He described the pain as sharp and brief, approaching an 8 on a 1‐10 scale. He denied any numbness in either upper extremity.

**FIGURE 1:**
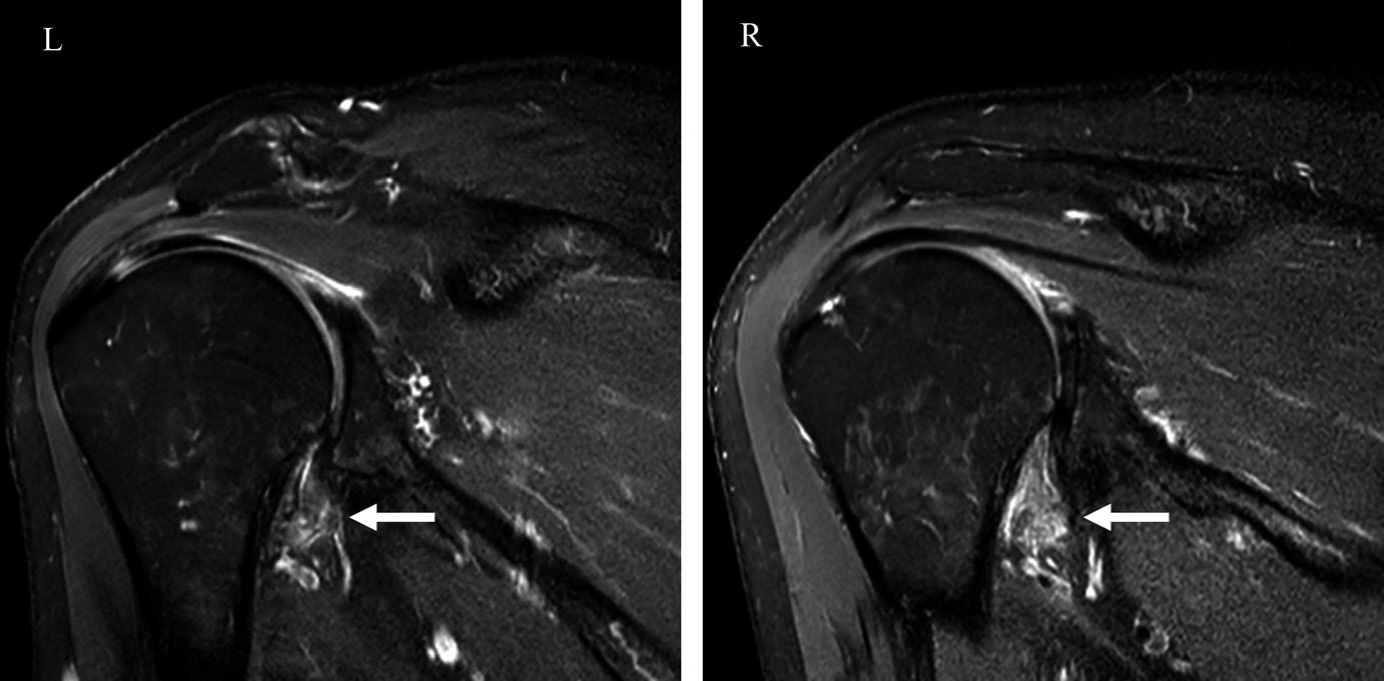
Magnetic resonance imaging of the bilateral shoulders depicting thickened and edematous inferior glenohumeral ligaments (white arrows). L, left; R, right

Physical examination of the right shoulder showed a range of motion of 115° flexion, 40° extension, 60° abduction, 90° internal rotation, and 40° external rotation. Left shoulder range of motion was 105° flexion, 40° extension, 60° abduction, 80° internal rotation, and 35° external rotation. The patient had positive Hawkins Signs and pain with acromioclavicular joint compression, bilaterally. Spurling signs were negative. Radiographs were unremarkable. Magnetic resonance imaging (MRI) showed bilateral thickening and edema of the inferior glenohumeral ligaments with loss of fat in the rotator interval, which is observed in adhesive capsulitis (Figure 1).

The patient was referred to physical therapy and had bilateral fluoroscopic‐guided glenohumeral corticosteroid injections of 1 mL of 40 mg/mL Depo‐Medrol and 4 mL 0.5% ropivacaine. Three months following the corticosteroid injections, the patient had marked improvements in shoulder range of motion and strength.

## 3 DISCUSSION

While reports have described shoulder‐related pain and dysfunction restricted to the limb in which the vaccination was administered, this is the first case to demonstrate that vaccinations may induce a systemic immune response resulting in bilateral adhesive capsulitis. Needle trauma related to the injection technique is unlikely to have caused the bilateral adhesive capsulitis as the limb contralateral to the injection was also involved. An immune‐mediated response to the influenza vaccination is believed to be the cause.

The exact immunological mechanism behind the pathogenesis of adhesive capsulitis is not well understood. Both synovial inflammation and joint capsular fibrosis are thought to contribute to adhesive capsulitis.^8^ Human leukocyte antigen B27 (HLA‐B27) may play a role in the pathogenesis.^8^ However, studies have found mixed associations between HLA‐B27 and adhesive capsulitis.^8^ Alternatively, it has been suggested that cytokines may be involved in the pathogenesis of adhesive capsulitis. Tumor necrosis factor α, interleukin 6, transforming growth factor β, and platelet‐derived growth factor have been found in capsular and synovial specimens of patients with adhesive capsulitis.^9^, ^10^ We are unable to comment on the exact mechanism of the immune response, but it is most likely that a systemic immune response to the administration of the vaccination resulted in the development of bilateral adhesive capsulitis.

While the physical examination and MRI findings of edema within inferior glenohumeral ligament are strongly consistent with adhesive capsulitis, other shoulder pathologies were considered. Arthritis and rotator cuff tears were ruled out by imaging. The patient‐reported history, as well as physical examination findings, was not consistent with bursitis or tendonitis. Parsonage‐Turner Syndrome was also considered since there was bilateral involvement. However, the physical examination did not identify signs or symptoms of weakness or sensory changes consistent with a diagnosis of Parsonage‐Turner Syndrome.

Importantly, the risk of SIRVA does not outweigh the benefit of vaccinations. However, when shoulder‐related adverse events do occur, they may not be localized to the upper extremity in which a vaccine was administered. Clinicians should consider SIRVA in the evaluation of patients with shoulder complaints. Furthermore, proper technique by injecting into the midpoint of the deltoid (approximately 2 inches below the acromion process) avoids vaccine administration into the shoulder capsule.

## CONFLICT OF INTEREST

None declared.

## AUTHOR CONTRIBUTIONS

AT: conducted the literature search on the topic and drafted the initial version of the manuscript. EE: collected clinical information and provided critical revision of the manuscript for intellectual content.
